# Visualizing the dental biofilm matrix by means of fluorescence lectin-binding analysis

**DOI:** 10.1080/20002297.2017.1345581

**Published:** 2017-07-09

**Authors:** Pune N. Tawakoli, Thomas R. Neu, Mette M. Busck, Ute Kuhlicke, Andreas Schramm, Thomas Attin, Daniel B. Wiedemeier, Sebastian Schlafer

**Affiliations:** ^a^ Clinic of Preventive Dentistry, Periodontology and Cardiology, Centre of Dental Medicine, University of Zurich, Zurich, Switzerland; ^b^ Department of River Ecology, Helmholtz Centre for Environmental Research – UFZ, Magdeburg, Germany; ^c^ Section for Microbiology, Department of Bioscience, Aarhus University, Aarhus, Denmark; ^d^ Statistical Services, Centre of Dental Medicine, University of Zurich, Zurich, Switzerland; ^e^ Department of Dentistry and Oral Health, Aarhus University, Aarhus, Denmark

**Keywords:** Confocal laser scanning microscopy, dental biofilms, extracellular polymeric substances, glycoconjugates, lectins

## Abstract

The extracellular matrix is a poorly studied, yet important component of dental biofilms. Fluorescence lectin-binding analysis (FLBA) is a powerful tool to characterize glycoconjugates in the biofilm matrix. This study aimed to systematically investigate the ability of 75 fluorescently labeled lectins to visualize and quantify extracellular glycoconjugates in dental biofilms. Lectin binding was screened on pooled supragingival biofilm samples collected from 76 subjects using confocal microscopy. FLBA was then performed with 10 selected lectins on biofilms grown *in situ* for 48 h in the absence of sucrose. For five lectins that proved particularly suitable, stained biovolumes were quantified and correlated to the bacterial composition of the biofilms. Additionally, combinations of up to three differently labeled lectins were tested. Of the 10 lectins, five bound particularly well in 48-h-biofilms: *Aleuria aurantia* (AAL), *Calystega sepiem* (Calsepa), *Lycopersicon esculentum* (LEA), *Morniga-G* (MNA-G) and *Helix pomatia* (HPA). No significant correlation between the binding of specific lectins and bacterial composition was found. Fluorescently labeled lectins enable the visualization of glycoconjugates in the dental biofilm matrix. The characterization and quantification of glycoconjugates in dental biofilms require a combination of several lectins. For 48-h-biofilms grown in absence of sucrose, AAL, Calsepa, HPA, LEA, and MNA-G are recommendable.

## Introduction

The two most prevalent oral diseases – caries and periodontitis – are caused by biofilms [1]. The extracellular matrix, a major biofilm component, plays a pivotal role in the development of these infections. It surrounds microbial cells and promotes bacterial adhesion and cohesion processes. It also hinders the diffusion of antimicrobial agents, provides mechanical stability, prevents desiccation, and serves as a nutrient reservoir [2,3]. Studying the composition and metabolism of the dental biofilm matrix is of the utmost importance for a thorough understanding of the processes involved in dental disease, and it may contribute to the development of new approaches to disease control.

Much effort has been spent on elucidating the involvement of extracellular glucans and fructans, polysaccharides consisting of either glucose or fructose moieties, in the caries process [2,4]. *Streptococcus mutans*, strongly associated with dental caries, releases glycosyltransferases (GtfB, C, and D) to synthetize glucans in the presence of sucrose [5,6]. In turn, the binding of GtfB to bacterial surfaces converts other bacteria to glucan producers [7], yielding a rigid matrix that serves as a scaffold for biofilm architecture and contributes to the creation and preservation of highly acidic niches [8,9].

In contrast, the presence and spatial distribution of other extracellular glycoconjugates in dental biofilms have received comparatively little attention. A few reports have employed the fluorescently labeled lectins concanavalin A and wheat germ agglutinin (WGA) to visualize sugar moieties in the matrix [10–12], but to date, no systematic analysis of the glycoconjugates present on the surface of bacterial cells and in the intercellular space has been conducted.

Fluorescence lectin-binding analysis (FLBA) is a powerful tool to visualize and quantify the glycoconjugate fraction of the extracellular matrix in a non-destructive way [13,14]. FLBA has been employed to study the matrix architecture in a variety of different biofilms, including environmental, industrial, and medical biofilms [15–23]. After an initial screening step where the binding of a large number of fluorescently labeled lectins to a particular type of biofilm is tested, selected lectins exhibiting high affinity to the biofilms in question are employed to visualize and quantify specific glycoconjugate motifs [24].

Therefore, the aims of the current study were: (1) to screen the binding of 75 fluorescently labeled lectins to pooled dental biofilm samples in order to identify a set of lectins that are generally useful for matrix staining in dental biofilms; (2) to test which of the lectins selected during the screening are suitable to visualize glycoconjugates in 48 h biofilms grown *in situ* in the absence of dietary carbohydrates; (3) to study the spatial distribution and quantify the biovolume of these lectins in 48 h biofilms from nine different subjects using confocal laser scanning microscopy (CLSM) and digital image analysis; and (4) to correlate lectin binding to the species composition of the biofilms, as determined by 16S rRNA gene sequencing.

## Methods

### Collection of pooled biofilm samples

Supragingival biofilms were carefully collected with a scaler (Deppeler SA, Rolle, Switzerland) from patients who attended the Center of Dental Medicine (University of Zurich). The study was approved by the Ethics Committee of Zurich (KEK-ZH-no. 2015–0468). The group consisted of patients with differences in oral hygiene, dietary regimens, and oral diseases. No selection criteria other than age (≥18 years) were applied. Altogether, biofilm samples from 76 patients were collected from buccal and lingual supragingival tooth sites to obtain a highly diverse sample containing a wide spectrum of possible glycoconjugates in dental biofilm. Samples were stored in 0.9% NaCl at 4°C for no longer than 3 days. Liquid was carefully discarded from all vials, and sedimented biofilms were pooled in a single tube. The pooled sample was then distributed onto 75 microscope slides (Permanox® Cell Culture Slides; Nalge Nunc International, Rochester, NY), all of which were fixed with 4% paraformaldehyde at room temperature (3 h) and then washed once with phosphate-buffered saline (PBS). Residual liquids were removed using absorption triangles (Aichele Medico GmbH, Basel, Switzerland). Slides were stored in a moist chamber at 4°C until experimental use. The supernatant of the residual pooled biofilm was discarded, fixation was performed as described above, and the sample was stored at −20°C.

### Collection of biofilms grown *in situ*

Nine healthy volunteers aged 21–41 years (*M*_age_ = 29.2 years; four males) were enrolled in the study. Pregnant or breast-feeding women, subjects suffering from chronic systemic disease, and subjects who had received anti-inflammatory or antimicrobial therapy within the past 6 months were excluded. None of the participants showed signs of periodontal disease or active carious lesions. The experimental protocol was approved by the Ethics Committee of Aarhus County (M-20100032), and informed consent was obtained from all participants.

A total of 94 biofilms were grown *in situ* on custom-made non-fluorescent glass slabs (4 mm × 4 mm × 1 mm; Menzel, Braunschweig, Germany) with a surface roughness of 1,200 grit. For each study participant, an individually designed lower jaw splint with buccal flanges was manufactured. Glass slabs were mounted at a slightly recessed position on each buccal flange to provide protection from shear forces. This *in situ* model is described in more detail by Dige et al. [25]. The study participants wore the splints for 48 h periods and only removed them from the mouth for oral hygiene maintenance and during intake of food or liquids other than water. After biofilm growth, the glass slabs were removed carefully from the splints and fixed for 3 h at 4°C in 4% paraformaldehyde in PBS. The glass slabs were then washed with PBS and stored in PBS/ethanol (1:1 v/v) at −20°C until further use.

### Screening of fluorescently labeled lectins

Each of the pooled biofilm samples was stained with 100 µL of one fluorescently labeled lectin (working concentration 100 µg/mL) for 30 min at room temperature [24]. Altogether, 65 fluorescein isothiocyanate (FITC) conjugated lectins and 10 Alexa Fluor® 488 conjugated lectins were used (Supplementary Data 1). Staining solutions were discarded using absorption triangles, and specimens were carefully washed three times with PBS. For microscopic analysis, specimens were placed upside down onto chamber slides (Nunc, ThermoFisher, Germany) filled with 100 µL of PBS. Based on visual examination of CLSM images, lectins showing strong fluorescence signals (voltage settings of the photomultiplier = 400–600 V) were chosen for subsequent analyses [26].

### FLBA of biofilms grown *in situ*

After the initial screening, the following 10 FITC-labeled lectins were tested on biofilms grown *in situ* from one subject in triplicate: *Aleuria aurantia* (AAL), *Agaricus bisporus* (ABA), *Allium sativum* (ASA), *Calystega sepiem* (Calsepa), *Helix pomatia* (HPA), *Lycopersicon esculentum* (LEA), *Morniga-*G (MNA-G), *Maclura pomifera* (MPA), *Pisum sativum* (PSA), and *Vicia graminea* (VGA). Additionally, *Helix aspersa* (HAA), which had shown weak binding during the screening, was used as a negative control. The bacteria were counterstained with SYTO® 60 (Molecular Probes; Thermo Fisher, Carlsbad, CA) according to the manufacturer’s instructions (incubation time 5 min). Lectin binding was assessed visually in CLSM images.

The biovolumes stained by five lectins showing strong binding in many areas of the biofilms (AAL, Calsepa, LEA, MNA-G, and HPA) were then quantified in biofilms from nine different subjects using CLSM and digital image analysis.

Furthermore, the following combinations of two or three different fluorescently labeled lectins were employed on four biofilms from one subject: (1) tetramethyl rhodamine isothiocyanate (TRITC)-labeled HPA, FITC-labeled MNA-G; counterstaining with SYTO® 60; (2) TRITC-labeled HPA, FITC-labeled VGA, Alexa Fluor® 647-labeled AAL; counterstaining with 4,6-diamidine-2-phenylindole (DAPI; 1 µg/mL; Sigma–Aldrich, Buchs, Switzerland); (3) TRITC-labeled HPA, FITC-labeled LEA, counterstaining with SYTO® 60; and (4) TRITC-labeled LEA, FITC-labeled VGA; counterstaining with SYTO® 60.

### CLSM

Pooled biofilm samples were examined with a TCS-SP1 CLSM (Leica Microsystems, Wetzlar, Germany) equipped with a 63× NA 1.2 water immersion objective. An Argon laser (488 nm) was used for excitation, and emission was detected with a photomultiplier between 500 and 600 nm (image size 512 × 512 pixels, line average = 1, pinhole size = 1 AU).

A TCS-SP5X CLSM (Leica Microsystems) with supercontinuum light source was used for assessment of *in situ* biofilms. The system was controlled by the software LAS AF v2.7.3.9723. Excitation/emission settings were as follows: DAPI – 405 nm/420–498 nm; FITC, Alexa Fluor® 488 – 490 nm/500–570 nm; TRITC – 555 nm/570-650nm; and SYTO® 60, Alexa Fluor® 647 – 650 nm/665–750 nm. High-quality imaging was performed using a 63× NA 1.2 water immersion objective. Z-stacks spanning the entire biofilm (step size 0.5 µm) were recorded in selected locations (image size 1,024 × 1,024 pixels, line average = 4, pinhole size = 1 AU, 400 Hz). Combinations of differently labeled lectins were imaged with single scans (line average = 48, pinhole size = 1 AU, 400 Hz). Prior to biofilm staining, the lectin combinations used were controlled visually for precipitate formation. For quantification of biovolumes stained with different lectins, a 25× NA 0.95 water immersion objective was used. Five z-stacks spanning the entire biofilm (step size 0.5 µm) were acquired in random locations of each specimen (image size 1,024 × 1,024 pixels, line average = 2, pinhole size = 1 AU).

### Digital image analysis

Image processing was performed with Imaris Software 8.3.1 (Bitplane, Zurich, Switzerland) after deconvolution using Huygens Remote Manager (Scientific Volume Imaging B.V. 2.1.2). Image data sets were quantified using the JImage Analyzer extension of ImageJ (http://rsbweb.nih.gov/ij). In each z-stack, the volumes stained with the labeled lectin and with SYTO® 60 were calculated. For subsequent analysis, biovolume ratios of lectin-specific glycoconjugates/bacteria were taken.

### 16S rRNA gene sequencing

For sequencing, *in situ*–grown biofilm samples and residual pooled biofilm were gently washed in DNA extraction buffer [27] to remove ethanol and PBS. Sample slabs were immersed in 300 µL of DNA extraction buffer [27] and subjected to enzymatic digestion with two different enzyme mixtures, as described by Juretschko et al. [27]. Subsequent steps of DNA extraction were performed using PowerLyzer® PowerSoil® DNA Isolation Kit (MoBio, Carlsbad, CA) according to the manufacturer’s protocol [28].

16S rRNA gene amplicons were prepared in accordance with Illumina’s 16S Metagenomic Sequencing Library Preparation guide [29] with a few modifications: variable regions V3 and V4 of bacterial 16S ribosomal RNA gene were amplified using primers Bac 341F and Bac 805R [30]. Three polymerase chain reaction (PCR) amplifications were carried out for each sample, the first amplifying the region between the primers (20 cycles), the second adding overhang adapters with modified primers (10 cycles), and finally an index PCR with Nextera XT index primers (Illumina, San Diego, CA) for sample identification (eight cycles). Each PCR had a total reaction volume of 25 µL containing 2.5 µL of DNA sample, 0.5 µL of each primer, 12.5 µL 2× KAPA HiFi HotStart Ready Mix (Kapa Biosystems, Wilmington, MA), and 9 µL of sterile H_2_O. The PCRs were performed on a Veriti® 96-well thermal cycler (Applied Biosystems, Waltham, MA) using the following cycle conditions: denaturation at 95°C for 3 min; cycles of 95°C, 55°C, and 72°C for 30 s each; and finally a 5 min extension step at 72°C. After each PCR, samples were purified using AMPure XP beads (Illumina). DNA concentration was measured on a Qubit fluorimeter (Thermo Fisher Scientific, Waltham, MA), and samples were diluted to approximately 3 ng DNA/µL. A pooled library containing indexed amplicons from all samples was sequenced on a MiSeq desktop sequencer (Illumina) using 2 × 300 bp chemistry (Illumina) according to the manufacturer’s instruction.

### 16S RRNA gene sequence analysis

Forward and reverse reads were assembled using PEAR 0.9.6 [31]. Unassembled reads and chimeras were discarded. The assembled data set was quality screened using fastQC software v0.9.6 [32], and a sequence length covering most of the data set was selected. Reads were trimmed to 400 bp and then filtered and clustered with usearch v8.1.1861 and the UPARSE pipeline [33]. Operational taxonomic units (OTUs) were generated on the basis of a high-quality subset of the data (maxee = 1.0), and the remaining reads were mapped onto these OTUs with a 97% similarity cutoff. OTUs were classified to genus level using mothur v1.36.1 [34], with the Silva SSU Ref NR release 123 database serving as a reference [35]. Further data analysis was done in R [36]. Data from the same patient were pooled (two samples each for subjects 1–8, one sample for subject 9). The data were normalized by rarefaction to a sampling depth of 18,000 OTUs per patient.

### Statistical analyses

Biovolumes ratios derived from the digital image analysis were further analyzed per subject and lectin. For each selected lectin (AAL, Calsepa, HPA, LEA, and MNA-G), the median of the biovolume ratios was calculated per subject over the respective z-stacks (Table 1). Significant differences between subjects were assessed using the Kruskal–Wallis test and *post hoc* pairwise comparisons according to Conover [37]. *p*-Values were corrected for multiple testing according to Benjamini–Hochberg. Moreover, glycoconjugate/bacteria ratios for each lectin were correlated to OTUs derived from the 16S rRNA gene analysis using Spearman correlation coefficients and the Benjamini–Hochberg correction. Only OTUs with a relative abundance >0.55% were included in the analysis. The significance level was set to α = 0.05, and all calculations were performed in R [36].

**Table 1.** Biovolumes stained by different fluorescently labeled lectins in biofilms grown in situ for 48-h in the absence of dietary carbohydrates. Bacterial and glycoconjugate biovolumes were quantified separately in five CLSM image stacks per subject and lectin. The median ratio of glycoconjugate/bacterial biovolume was calculated for all investigated microscopic fields of view. Median and interquartile range (IQR) were then calculated for each subject and lectin. Moreover, the median and IQR of each lectin were calculated over all subjects. N/A = excluded due to unreliable staining.

Subject	*Aleuria aurantia*	*Lycopersicon esculentum*	*Calystega sepiem*	*Morniga*-G	*Helix pomatia*
1	1.97 (1.27)	1.05 (0.08)	1.12 (0.19)	3.84 (15)	0.17 (0.16)
2	0.17 (0.09)	2.42 (1.83)	1.15 (0.84)	0.19 (0.43)	0.51 (0.31)
3	6.84 (4.76)	1.62 (0.19)	1.46 (1.19)	1.81 (1.01)	0.20 (0.07)
4	6.47 (9.91)	1.59 (1.75)	2.71 (1.08)	5.57 (5.19)	1.38 (1.04)
5	0.55 (0.50)	1.09 (0.91)	0.10 (0.12)	0.52 (0.31)	0.12 (0.05)
6	0.36 (0.23)	0.70 (0.67)	0.30 (1.49)	0.26 (0.16)	0.04 (0.01)
7	2.66 (0.50)	0.75 (0.35)	0.30 (0.10)	7.98 (4.53)	1.33 (0.22)
8	2.81 (7.62)	0.37 (0.51)	N/A	1.24 (0.43)	0.10 (0.02)
9	5.35 (4.81)	0.93 (0.25)	0.51 (0.17)	1.24 (0.43)	0.07 (0.01)
Median (IQR)	2.66 (4.8)	1.05 (0.84)	0.81 (0.92)	1.81 (3.32)	0.17 (0.41)

## Results

### FLBA of pooled biofilm samples and biofilms grown *in situ*

**Figure 1.**
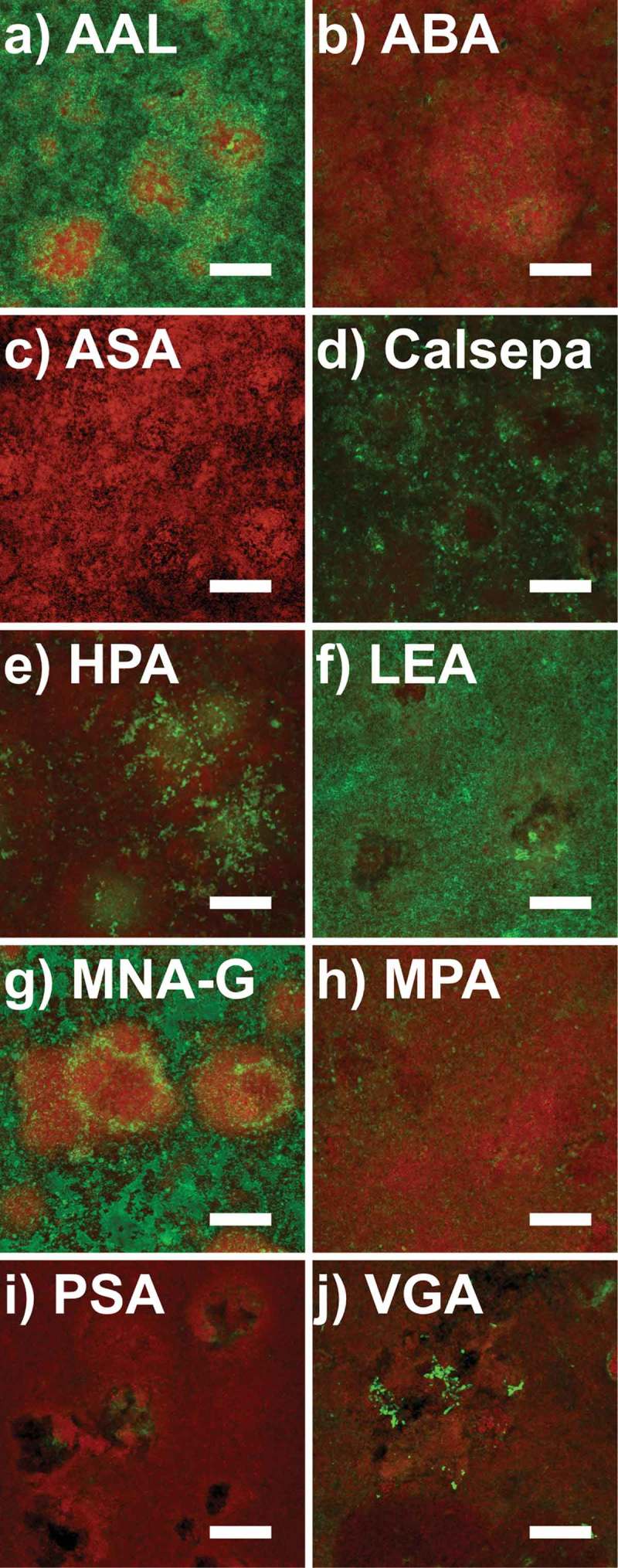
Confocal laser scanning microscopy (CLSM) images of 48 h biofilms grown *in situ* stained with fluorescently labeled lectins. Panels a–j show maximum intensity projections of biofilm images from one subject, with the FITC-labeled lectins in green and the nucleic acid stain SYTO® 60 in red. *Aleuria aurantia* (AAL), *Calystega sepiem* (Calsepa), *Lycopersicon esculentum* (LEA), and *Morniga*-G (MNA-G) show strong fluorescence signals. *Helix pomatia* (HPA) shows strong selective binding inside dense bacterial clusters. *Vicia graminea* (VGA) binds selectively to bacterial surfaces in branched spider web-like colonies. *Agaricus bisporus* (ABA), *Allium sativum* (ASA), *Maclura pomifera* (MPA), and *Pisum sativum* (PSA) show rather diffuse or no signals at all. Scale bars = 25 µm.

**Figure 2.**
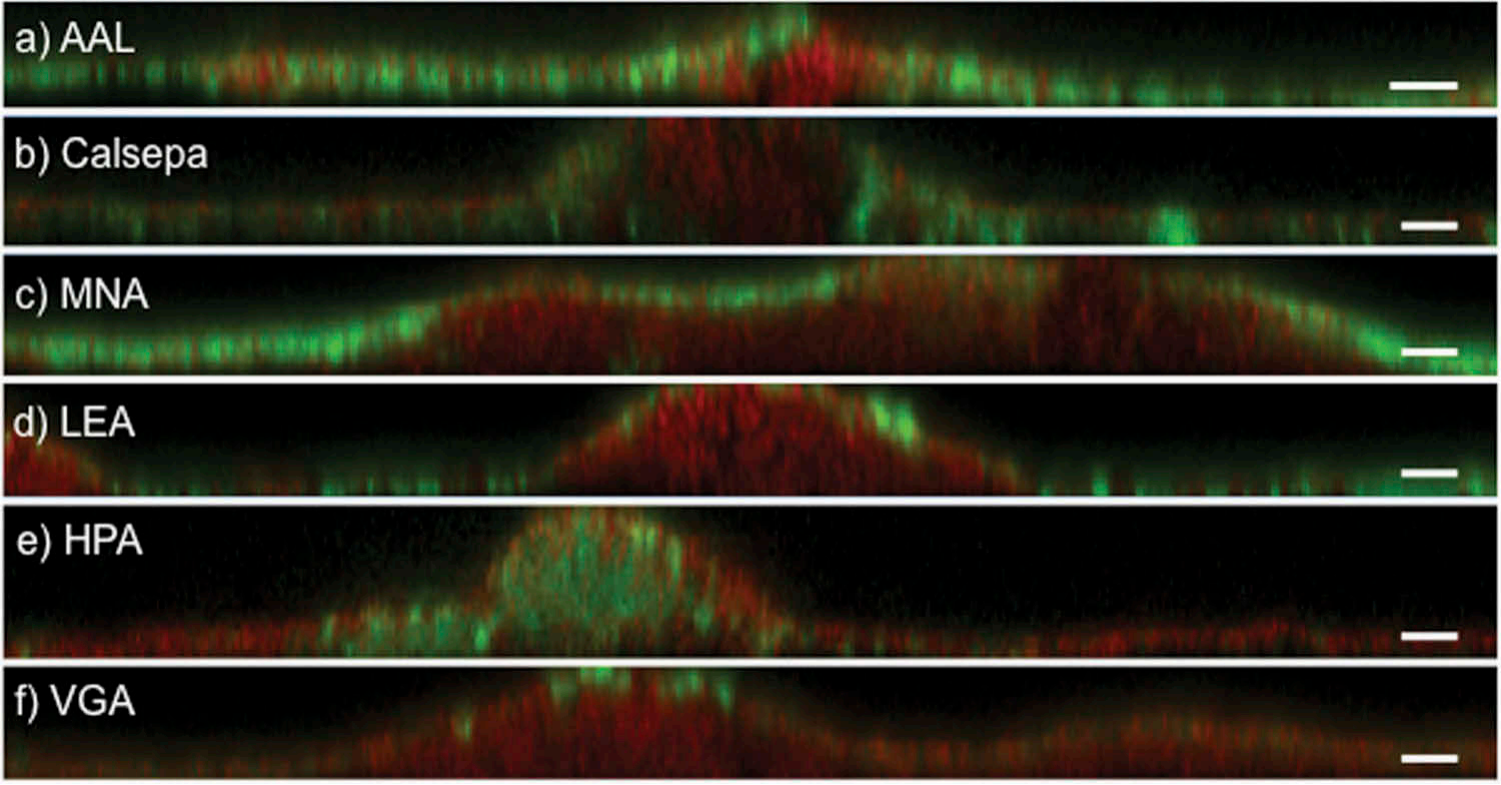
Deconvoluted CLSM images of 48 h biofilms grown *in situ* stained with fluorescently labeled lectins. Panels a–f show representative xz projections of lectin-stained biofilms (green). AAL, Calsepa, LEA, and MNA-G bind on the biofilm surface and in thin areas of the biofilms. HPA binds selectively to dense cell clusters in protuberations of the biofilms. VGA binds selectively to the surface of some organisms. Bacterial staining was performed with SYTO® 60 (red). Scale bars = 5 µm.

During the initial screening of lectin binding in pooled biofilm samples, 41 lectins showed weak or no fluorescence signals (data not shown), 24 lectins showed moderate fluorescence signals (Supplementary Data 2), while 10 lectins (AAL, ABA, ASA, Calsepa, HPA, LEA, MNA-G, MPA, PSA, and VGA) yielded strong fluorescence signals in the pooled samples (Supplementary Data 1 and 3) and were further tested on biofilms grown *in situ* for 48 h in the absence of dietary carbohydrates (Figure 1). In these biofilms, five lectins (AAL, Calsepa, HPA, LEA, and MNA-G) showed strong fluorescence signals in either large or confined areas of the matrix (Figure 2). HPA penetrated well into dense bacterial colonies within mushroom-like protuberations of the biofilms, whereas AAL, Calsepa, LEA, and MNA-G preferentially bound to the outside of dense cell clusters and in areas with lower cell density. VGA bound very selectively to cell surface glycoconjugates of branched, spider web-like colonies. ABA, ASA, MPA, PSA, and the negative control HAA showed only very weak or no fluorescence signals (Figure 1).

### Quantification of lectin-stained biovolumes

For quantification, lectin-stained biovolumes were normalized to the respective bacterial biovolumes. AAL stained the largest relative biovolume with a median of 2.66 (interquartile range [IQR] = 4.8), followed by MNA-G (1.81; IQR = 3.32), LEA (1.05; IQR = 0.84), Calsepa (0.81; IQR = 0.92), and HPA (0.17; IQR = 0.41). Differences were statistically significant between AAL and HPA (*p* = 0.009) and MNA-G and HPA (*p* = 0.015).

The relative biovolumes stained by particular lectins differed largely between patients. For example, AAL-stained biovolumes ranged from 0.17 (IQR = 0.09) to 6.84 (IQR = 4.76), and MNA-G-stained biovolumes from 0.19 (IQR = 0.43) to 7.98 (IQR = 4.53). For AAL, LEA, and MNA-G, the total lectin-stained biovolume was bigger than the bacterial biovolume, while Calsepa- and especially HPA-stained biofilms exhibited higher bacterial biovolumes than lectin-stained biovolumes. (See Table 1 for comprehensive data.)

### Correlation between lectin binding and bacterial biofilm composition

**Figure 3.**
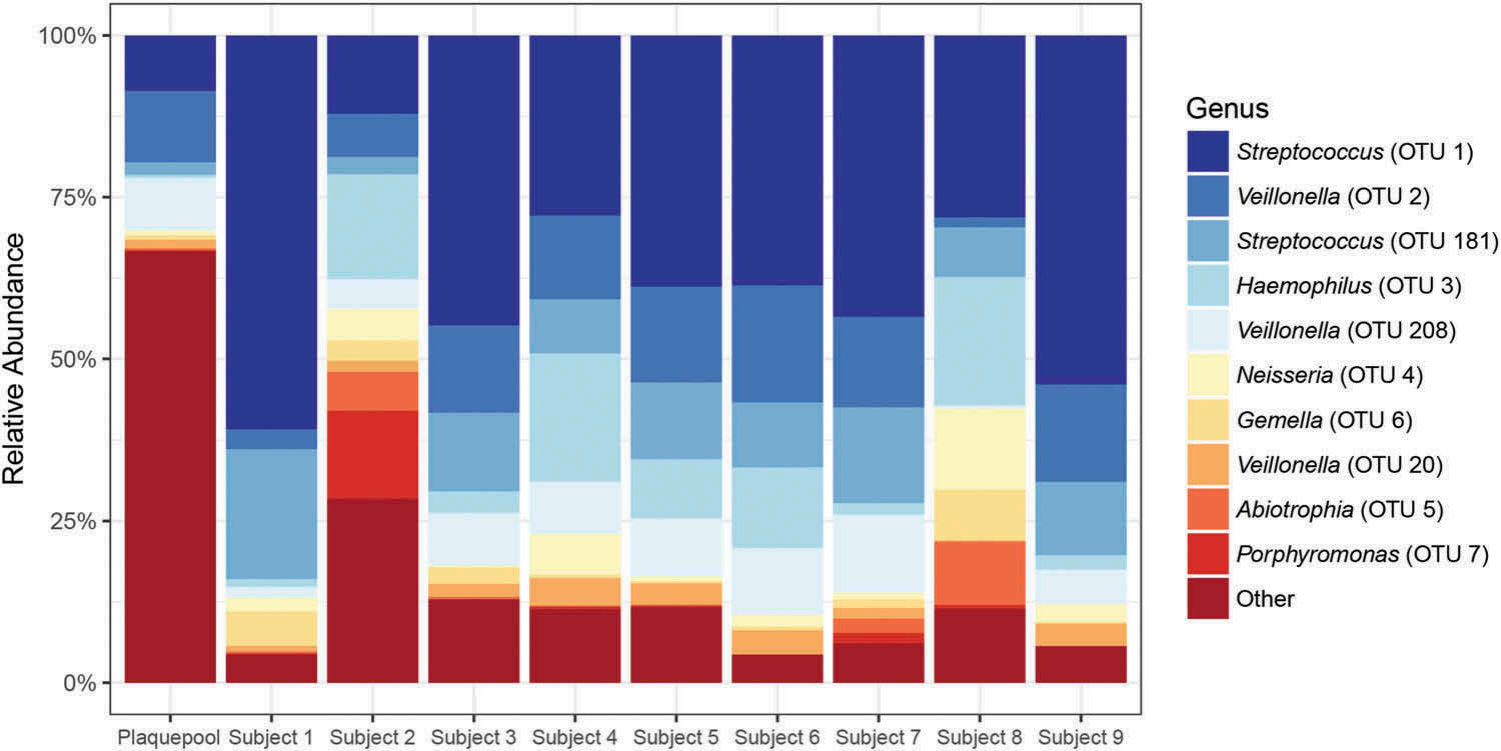
Relative abundances of bacterial operational taxonomic units (OTUs) in dental biofilm samples classified to genus level. The 10 most abundant OTUs are shown; all other OTUs are pooled. When the same genus name is represented more than once, the 16S rRNA gene fragment sequences were indicative of multiple species (<97% identical). The pooled biofilm sample showed higher species richness than the 48 h biofilms from subjects 1–9. All biofilms were dominated by *Streptococcus* and *Veillonella.*

16S rRNA gene sequence analysis identified a total of 207 different OTUs across all samples. The pooled biofilms used for the initial lectin screening showed greater taxonomic richness compared to the 48 h biofilms (Supplementary Data 4). Pooled biofilms harbored predominantly *Streptococcus*, *Veillonella*, and *Fusobacterium* spp., but also members of the genera *Corynebacterium*, *Leptotrichia, Actinomyces*, *Capnocytophaga, Campylobacter*, *Lachnoanaerobaculum, Dialister*, *Olsenella*, and *Lactobacillus*. Forty-eight-hour biofilms consisted mainly of *Streptococcus* and *Veillonella* spp.; members of the genera *Haemophilus*, *Neisseria, Gemella*, *Abiotrophia, Fusobacterium*, *Alloprevotella*, and *Actinomyces* were also present. Figure 3 shows the relative abundances of the 10 most abundant OTUs. Fifty-six OTUs with a relative abundance >0.55% were included in the correlation analysis with lectin biovolumes. Benjamini–Hochberg correction-adjusted analysis, however, showed no significant correlations between the binding of particular lectins and the abundance of specific OTUs.

### Combined application of different lectins

**Figure 4.**
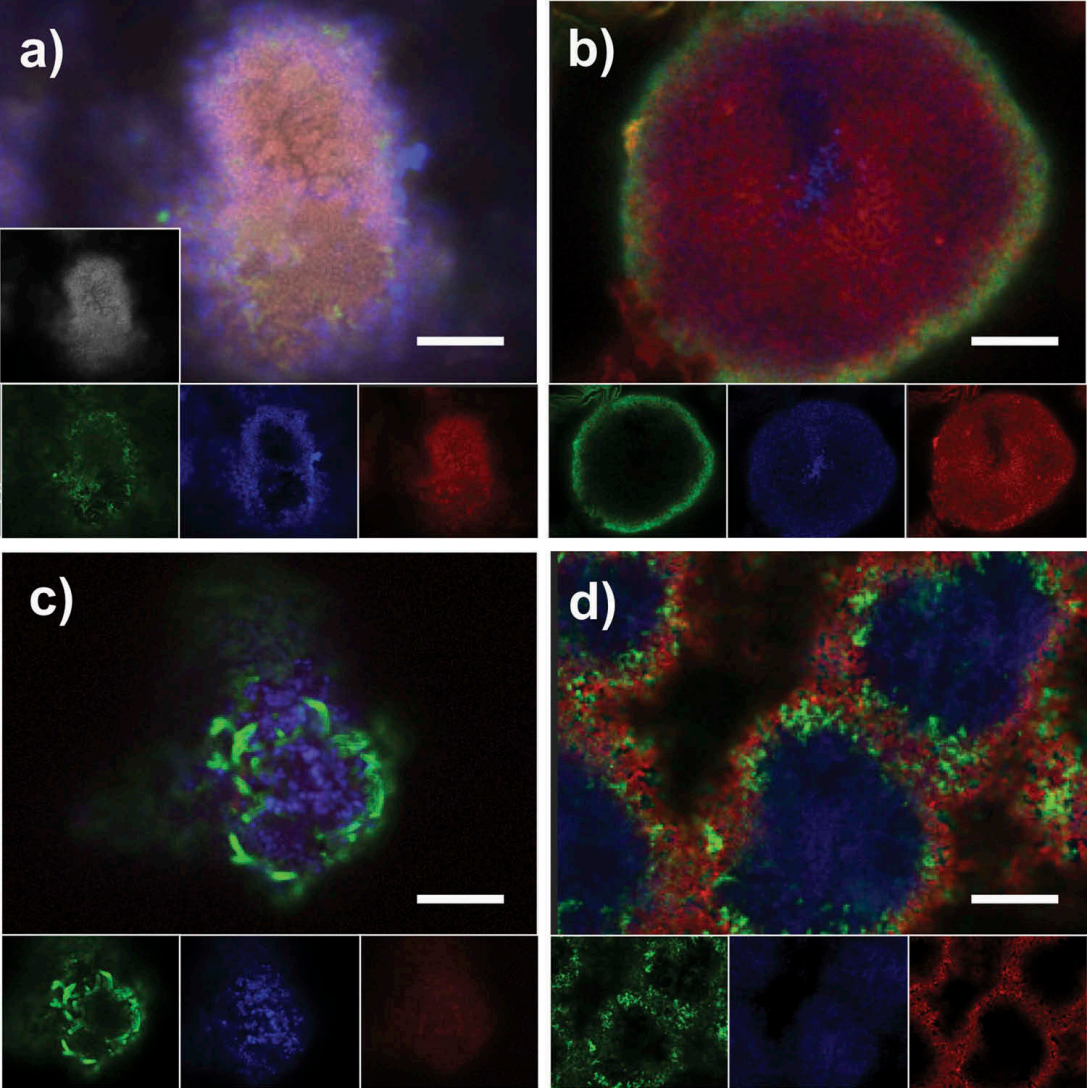
CLSM images of 48 h biofilms grown *in situ* stained with combinations of fluorescently labeled lectins. Panels a–d show the overlay and single channel images of triple and quadruple-channel biofilm scans from one subject, illustrating the binding patterns of (a) AAL-Alexa Fluor® 647 (blue), HPA-TRITC (red), and VGA-FITC (green) combined with DAPI (gray); (b) LEA-FITC (green) and HPA-TRITC (red) combined with SYTO® 60 (blue); (c) LEA-TRITC (red), VGA-FITC (green) in combination with SYTO® 60 (blue); and (d) MNAG-FITC (green) and HPA-TRITC (red) combined with SYTO® 60 (blue). Scale bars = 10 µm.

In most cases, the combined application of differently labeled lectins with a nucleic acid stain resulted in the same binding pattern as observed for single use: FITC-labeled VGA, Alexa Fluor® 647-labeled AAL, and TRITC-labeled HPA in combination with DAPI (Figure 4a), as well as FITC-labeled LEA and TRITC-labeled HPA in combination with SYTO® 60 stained the same areas of the biofilms as observed during separate application (Figure 4b). Likewise, FITC-labeled VGA and TRITC-labeled LEA in combination with SYTO® 60 bound to areas comparable with individual application, albeit with weaker signals for LEA (Figure 4c). In contrast, when TRITC-labeled HPA was employed with FITC-labeled MNA-G and SYTO® 60, HPA bound only to the outer layers of dense cell clusters (Figure 4d), while it showed penetration into the cluster core when applied individually (Figure 1).

## Discussion

Studying the presence and spatial distribution of glycoconjugates in the dental biofilm matrix is an important step toward an in-depth understanding of biofilm architecture and physiology. A few studies have used fluorescently labeled lectins to map matrix glycoconjugates [15–22], but the present study is the first to perform a systematic analysis of the suitability of different lectins for matrix visualization in dental biofilms. The binding of 75 different commercially available lectins were screened using pooled biofilm samples collected from buccal and other oral sites in a large number of patients (*n* = 76). No further standardization of the samples was performed in order to obtain a highly diverse pooled sample.

The screening identified 10 lectins (AAL, ABA, ASA, Calsepa, HPA, LEA, MNA-G, MPA, PSA, and VGA) that yielded strong fluorescence signals, thus being particularly suitable for the characterization of glycoconjugates in the dental biofilm matrix. Interestingly, application of the most frequently employed fluorescently labeled lectins, concanavalin A and WGA, did not result in particularly strong fluorescence signals in pooled biofilms (screening scores ± and +, respectively; Supplementary Data 1). Their use for the visualization of glycoconjugates in the dental biofilm matrix should therefore be reconsidered.

The 10 selected lectins were then applied on biofilms grown *in situ* for 48 h in a caries-free subject and without any exposure to dietary carbohydrates. In these highly standardized biofilms, only 5/10 lectins (AAL, Calsepa, HPA, LEA, and MNA-G) yielded strong and widespread fluorescence signals, which likely reflects the presence of a smaller set of extracellular glycoconjugates compared to the pooled biofilm sample. It should be noted that the binding of the 10 lectins and the negative control HAA was evaluated in 33 replicate biofilms derived from one subject and that inter-individual variation therefore was not taken into account. Quantification of the stained biovolumes in biofilms from nine subjects, however, showed that all five lectins reliably stained matrix glycoconjugates with reproducible binding patterns. HPA, with a binding specificity for N-acetylgalactosamine, was the only lectin to penetrate and bind preferentially in dense bacterial clusters in mushroom-like protuberations of the biofilms, whereas AAL, Calsepa, LEA, and MNA-G, with specific affinities for fucose, mannose, N-acetylglucosamine, and galactose, respectively, stained glycoconjugates on the biofilm surface and in thin or less-dense parts of the biofilms (Figure 2). As many areas of the investigated biofilms were thin (10–25 µm thickness), the latter four lectins stained larger biovolumes compared to HPA, with AAL and MNA-G being most prevalent. The sole use of AAL and HPA, however, is not recommended for a comprehensive visualization of matrix glycoconjugates, since the stained biovolumes differed considerably between subjects for all employed lectins (see Table 1). Based on these results, the use of all five lectins is recommended for the analysis of young dental biofilms grown in the absence of dietary carbohydrates. However, investigations on matrix glycoconjugates in older biofilms, biofilms from caries-active subjects, and, in particular, in biofilms grown in the presence of sucrose [38–40] should start by screening the suitability of the 10 most suitable lectins prior to FLBA.

The 48 h biofilms in the present study were not grown on dental enamel but on non-fluorescent glass surfaces in order to facilitate confocal microscopy analysis. Previous studies have shown that while the surface roughness is of crucial importance for biofilm growth [41], the material of the underlying substrate has less influence on biofilm architecture due to masking effects by the salivary coating [42].

The present study shows that mono- or disaccharide specificities, which are frequently used to group lectins, do not allow predictions of lectin binding to a particular biofilm sample. Both HAA and HPA, for example, bind N-acetylgalactosamine and, with lower affinity, N-acetylglucosamine. While HPA showed excellent binding in both the pooled biofilms and the 48 h biofilms, HAA was not able to bind to any of the investigated samples and consequently was chosen as a negative control. ASA, Calsepa, and PSA share a high affinity for mannose, and the latter two both recognize glucose moieties, but of the three lectins, only Calsepa bound well in the 48 h biofilms (Figure 1).

Lectin binding can be inhibited competitively by monosaccharides, but often, the binding affinity to complex glycoconjugates is an order of magnitude higher, and binding is not only determined by the steric structure of particular oligosaccharide chains but may also be influenced by the polypeptide backbone of the glycoproteins [43,44]. The preferential binding of VGA to clusters of O-linked galactose (β1-4) N-acetylgalactosamine adjacent to N-terminal leucine residues may serve as an example. Furthermore, the lectin-mediated visualization of matrix glycoconjugates depends on other factors, such as biofilm penetration (which again is influenced by molecular weight, the isoelectric point, and the net charge of a lectin), the used fluorochromes, and, if applied in combination, the interaction between different lectins and the order of application [14]. The present results showed that TRITC-labeling of LEA resulted in weaker fluorescence signals compared to FITC-labeling, and that TRITC-labeled HPA was not able to penetrate dense cell clusters in the biofilms when applied in combination with FITC-labeled VGA. Overall, FITC-conjugated lectins have proven to yield reliable staining results in biofilms [14].

16S rRNA gene sequencing revealed that the species composition of the biofilm samples was similar to the one found in previous studies [45–47], with a predominance of *Streptococcus* and *Veillonella* and a limited number of other genera. The species richness in the 48 h biofilms was considerably lower than in the pooled biofilm sample (Figure 3 and Supplementary Data 4), confirming the intended high bacterial diversity of the biofilms used for the initial screening. As the biovolumes stained by individual lectins in the 48 h biofilms differed considerably between subjects, a correlation analysis was performed to identify possible relationships between species abundance and the presence of particular matrix glycoconjugates. However, no correlations between lectin binding and specific OTUs were found. To uncover the relationship between certain matrix glycoconjugates and their producers, a CLSM-based co-localization approach that combines FLBA and fluorescence *in situ* hybridization (FISH) with species- or genus-specific probes might be more promising. VGA, for example, which was not included in the quantitative analysis, showed a very specific binding to clusters of bacteria with a cell and colony morphology that possibly represented *Actinomyces* spp. (Figure 4c). A spatial correlation analysis in combined FISH/lectin stained images might confirm this hypothesis in further studies.

## Conclusion

Fluorescently labeled lectins are powerful tools to visualize and quantify extracellular glycoconjugates in dental biofilms. The lectins AAL, Calsepa, HPA, LEA, and MNA-G are particularly suitable for identifying glycoconjugates in 48 h biofilms grown *in situ* in the absence of dietary carbohydrates. Multiple fluorescently labeled lectins may be used in combination, but changes in the binding pattern cannot be excluded.

## Supplementary Material

Supplemental_data.zip
