# Supplementary material for: Visualizing the dental biofilm matrix by means of fluorescence lectin-binding analysis
Source: J Oral Microbiol. 2017 Jul 9;9(1):1345581. doi: 10.1080/20002297.2017.1345581 (PMC5508396; doi:10.1080/20002297.2017.1345581)
Supplement: Supplemental_data.zip [file zjom_a_1345581_sm2449.zip › Supplemental data/Suppl__1.docx]

Table S1. **Suitability of different fluorescently labeled lectins for visualization of extracellular glycoconjugates in dental biofilm**.

| **Lectin (Conjugate)** | **Fluorescence signal** | **Specificity** |
| --- | --- | --- |
| **AAA**_*Anguilla anguilla* (FITC)^1^ | **-** | α-L-Fucose |
| **ABA**_*Agaricus bisporus* (FITC)^1^ | **++** | Galactose (β1-3) N-Acetylgalactosamine |
| **AIA**_*Artocarpus integrifolia* (FITC)^1^ | **+** | α-Galactose |
| **AAL**_*Aleuria aurantia* (FITC)^1,2^ | **++** | Fucose (α1-6) N-Acetylglucosamine, Fucose (α1-3) N-Acetyllactosamine structures, Fucose attached to nucleic acids |
| **AMA**_*Arum maculatum* (FITC)^1^ | **+** | Mannose |
| **ASA**_*Allium sativum* (FITC)^1^ | **++** | Mannose |
| **Ban**_*Musa paradisiaca* (FITC)^2^ | **+/-** | (α1-3) Glucose and Mannose, (β1-3) and (β1-6) Glucosyl-structures |
| **BDA**_*Bryonia dioica* (FITC)^1^ | **+/-** | N-Acetylgalactosamine |
| **BPA**_*Bauhinia purpurea* (FITC)^1^ | **+** | N-acetyl-D-Galactosamine |
| **CA**_*Colchicum autumnale* (FITC)^1^ | **+** | N/A |
| **CAA**_*Caragana aborescens* (FITC)^1^ | **+** | N-Acetylgalactosamine |
| **Calsepa**_*Calystega sepiem* (FITC)^1^ | **++** | Maltose = Mannose >> Glucose |
| **ConA**_*Canavalia ensiformis* (FITC)^1^ | **+/-** | α-D-Mannose, α-D-Glucose, branched Mannose |
| **CPA**_*Cicer arietinum* (FITC)^1^ | **+/-** | N/A |
| **CSA**_*Cytisus sessilifolius* (FITC)^1^ | **+** | N-Acetylgalactosamine |
| **DBA**_*Dolichos biflorus* (FITC)^3^ | **+/-** | Methyl-2-Acetamido-2-Deoxy-D-Galactose |
| **DGL**_*Dioclea grandiflora* (FITC)^1^ | **+/-** | Mannose, Glucose, Mannose trimers |

| **DSA**_*Datura* *stramonium* (FITC)^1^ | **+** | (β1-4) N-Acetylglucosamine oligomers |
| --- | --- | --- |
| **ECA**_*Erythrina cristagalli* (FITC)^1^ | **+** | Galactose (β1-4) N-Acetylglucosamine |
| **EEA**_*Euonymus europaeus* (FITC)^1^ | **+/-** | D-Galactose (α1-3) L-Fucose (α1-2) D-Galactose |
| **GHA**_*Glechoma hederacea* (FITC)^1^ | **-** | D-Galactose, Methyl α-D-Galactopyranoside, N-Acetylgalactosamine |
| **GNA**_*Galanthus nivalis* (FITC)^1^ | **+** | Mannose |
| **GS-I**_*Griffonia simplicifolia* (FITC)^3^ | **+/-** | Melibiose, α-D-Galactose |
| **HAA**_*Helix aspersa* (FITC)^1^ | **-** | N-Acetylgalactosamine |
| **HHA**_*Hippeastrum hybrid* (Amaryllis) (FITC)^1^ | **+** | Mannose (internal and terminal Mannose residues) |
| **HMA**_*Homarus americanus* (FITC)^1^ | **+/-** | Sialic acid (derivatives of N-Acetylneuraminic acid and N-Glycolylneuraminic acid) |
| **HPA**_*Helix pomatia* (FITC)^3^ | **++** | N-Acetylgalactosamine |
| **IRA**_*Iris hybrid* (FITC)^1^ | **-** | N-Acetyl- D-Galactosamine |
| **LAA**_*Laburnum alpinum* (FITC)^1^ | **+/-** | N-Acetylglucosamine (β1-4) N-Acetylglucosamine |
| **LAL**_*Laburnum anagyroides* (FITC)^1^ | **+/-** | α-Methyl-L-Fucose |
| **LBA**_*Phaseolus lunatus* (FITC)^1^ | **+/-** | N-Acetylgalactosamine (α1-3) L-Fucose (α1-2) D-Galactose |
| **LcH**_*Lens culinaris* (FITC)^1^ | **+** | N/A |
| **LEA**_*Lycopersicon esculentum* (FITC)^3^ | **++** | (β1-4) N-Acetylglucosamine |
| **LFA**_*Limax flavus* (FITC)^1^ | **-** | Sialic acid (derivatives of N-Acetylneuraminic acid and N-Glycolylneuraminic acid) |
| **Lotus**_*Tetragonolobus purpurea* (FITC)^3^ | **+/-** | L-Fucose |
| **LPA**_*Limulus polyphemus* (FITC)^1^ | **+** | N-Acetylneuraminic acid |
| **MAA**_*Maackia amurensis* (FITC)^1^ | **+** | N-Acetylneuraminic acid (α2-3) Galactose |
| **MNA-G**_*Morniga G* (FITC)^1^ | **++** | Galactose |

| **MOA**_*Marasmium* *oreades agglutinin* (FITC)^1^ | **+/-** | D-Galactose (α1-3) D-Galactose, D-Galactose (α1-3) D-Galactose (β1-4) N-Acetylglucosamine |
| --- | --- | --- |
| **MPA**_*Maclura pomifera* (FITC)^1^ | **++** | N-Acetylgalactosamine > Galactose |
| **NPA**_*Narcissus pseudonarcissus* (FITC)^1^ | **+** | Mannose |
| **PHA-E**_*Phaseolus vulgaris* (FITC)^1^ | **-** | PHA-E recognizes a complex branched chain oligosaccharide structure |
| **PHA-L**_*Phaseolus vulgaris* (FITC)^1^ | **+** | PHA-L recognizes a complex branched chain oligosaccharide structure |
| **PMA**_*Polygonatum multiflorum* (FITC)^1^ | **-** | Asialofetuin and Asialomucin >> native Fetuin and Mucin |
| **PNA**_*Arachis hypogaea* (FITC)^1^ | **-** | Terminal β-Galactose |
| **PSA**_*Pisum sativum* (FITC)^3^ | **++** | α-Mannose > α-Glucose |
| **PSL**_*Polyporus squamosus* (FITC)^1^ | **-** | N-Acetylneuraminic acid (α2-6) D-Galactose, (β1-4) N-Acetylglucosamine |
| **PTA**_*Psophocarpus tetragonolobus* (FITC)^3^ | **+/-** | N-Acetylgalactosamine, D-Galactose |
| **PWA**_*Phytolacca americana* (FITC)^1^ | **+** | N-Acetylglucosamine (β1-4) N-Acetylglucosamine oligomers, [D-Galactose (β1-4) N-Acetylglucosamine]_2_ |
| **RPA**_*Robinia pseudoaccacia* (FITC)^1^ | **+** | N/A |
| **SBA**_*Glycine max* (FITC)^1^ | **-** | α and β- N-Acetylgalactosamine > α and β-Galactose |
| **SJA**_*Sophora japonica* (FITC)^1^ | **+/-** | N-Acetylgalactosamine |
| **SNA**_*Sambucus nigra* (FITC)^1^ | **+/-** | N-Acetylneuraminic acid (α2-6) D-Galactose/ N-Acetylgalactosamine |
| **SSA**_*Salvia Sclarea* (FITC)^1^ | **+/-** | N-Acetylgalactosamine. Terminal N-Acetylgalactosamine linked to Serine (or Threonine) |
| **STA**_*Solanum tuberosum* (FITC)^1^ | **+** | N-Acetylglucosamine (β1-4) N- Acetylglucosamine oligomers |
| **TKA**_*Trichosanthes kirilowii* (FITC)^1^ | **+/-** | Galactose |
| **TL**_*Tulipa sp.* (FITC)^1^ | **+/-** | N-Acetylgalactosamine, Galactose, Fucose |
| **UDA**_*Urtica dioica* (FITC)^1^ | **+** | N-Acetylglucosamine |

| **UEA-I**_*Ulex europaeus* (FITC)^3^ | **+** | α-L-Fucose |
| --- | --- | --- |
| **VFA**_*Vicia faba* (FITC)^1^ | **+** | Mannose |
| **VGA**_*Vicia graminea* (FITC)^1^ | **++** | O-linked D-Galactose (β1-3) N-Acetylgalactosamine adjacent to an N-terminal Leucine residue |
| **VRA**_*Vigna radiata* (FITC)^1^ | **+/-** | α-Galactose |
| **VVA_***Vicia villosa* (FITC)^1^ | **+/-** | N-Acetylgalactosamine |
| **WFA**_*Wisteria floribunda* (FITC)^1^ | **+/-** | N-Acetylgalactosamine |
| **WGA**_*Triticum vulgaris* (FITC)^3^ | **+** | (N-Acetylglucosamine)2, N-Acetylneuraminic acid |
| **CCA**_*Cancer antennarius crude* (Alexa Fluor® 488)^1^ | **--** | N/A |
| **Co**_*Codium fragile* (Alexa Fluor® 488)^3^ | **-** | N/A |
| **IAA**_*Iberis amara* (Alexa Fluor® 488)^1^ | **+** | N/A |
| **MIA**_*Mangifera indica crude*  (Alexa Fluor® 488)^1^ | **-** | N/A |
| **PAA**_*Perseau americana crude*  (Alexa Fluor® 488)^1^ | **-** | N/A |
| **PA-I**_*Pseudomonas aeruginosa*  (Alexa Fluor® 488)^3^ | **+** | N/A |
| **PPA**_*Ptilota plumosa* (Alexa Fluor® 488)^3^ | **-** | N/A |
| **RTA**_*Trifolia repens crude*  (Alexa Fluor® 488)^1^ | **-** | N/A |
| **SHA**_*Salvia hormonium crude*  (Alexa Fluor® 488)^1^ | **-** | N-Acetylgalactosamine |
| **SSC**_*Sarothamnus scoparius crude*  (Alexa Fluor® 488)^1^ | **-** | N/A |

Suitability was determined by visual inspection of CLSM images: ++ strong fluorescence signal, + moderate signal, +/- weak signal, - very weak signal, and -- no fluorescence signal.Specificities indicated as listed in the suppliers’ product data sheets (Suppliers: 1= E.Y. Laboratories Immunology, San Mateo, CA, USA; 2= Vector Laboratories, Burlingame, CA, USA; 3= Sigma-Aldrich, Buchs, Switzerland). N/A= Lectins without specificity allegation.
