# Supplementary material for: Visualizing the dental biofilm matrix by means of fluorescence lectin-binding analysis
Source: J Oral Microbiol. 2017 Jul 9;9(1):1345581. doi: 10.1080/20002297.2017.1345581 (PMC5508396; doi:10.1080/20002297.2017.1345581)
Supplement: Supplemental_data.zip [file zjom_a_1345581_sm2449.zip › Supplemental data/Suppl__4.docx]

| Genus | Plaquepool | Subject 1 | Subject 2 | Subject 3 | Subject 4 | Subject 5 | Subject 6 | Subject 7 | Subject 8 | Subject 9 |
| --- | --- | --- | --- | --- | --- | --- | --- | --- | --- | --- |
| *Streptococcus* (OTU 1) | 1568 | 10979 | 2117 | 8056 | 5052 | 7078 | 6980 | 7963 | 5016 | 9673 |
| *Veillonella* (OTU 2) | 1926 | 557 | 1254 | 2385 | 2329 | 2670 | 3197 | 2476 | 283 | 2718 |
| *Streptococcus* (OTU 181) | 352 | 3675 | 499 | 2188 | 1529 | 2136 | 1836 | 2662 | 1393 | 2046 |
| *Haemophilus* (OTU 3) | 85 | 206 | 2899 | 598 | 3498 | 1671 | 2311 | 304 | 3548 | 398 |
| *Veillonella* (OTU 208) | 1516 | 297 | 831 | 1492 | 1480 | 1550 | 1862 | 2152 | 95 | 972 |
| *Neisseria* (OTU 4) | 120 | 363 | 865 | 49 | 1125 | 131 | 316 | 190 | 2276 | 486 |
| *Gemella*  (OTU 6) | 125 | 917 | 538 | 467 | 98 | 56 | 99 | 223 | 1406 | 56 |
| *Veillonella* (OTU 20) | 249 | 158 | 322 | 341 | 747 | 602 | 642 | 246 | 33 | 613 |
| *Abiotrophia* (OTU 5) | 48 | 33 | 1111 | 64 | 31 | 28 | 1 | 396 | 1760 | 10 |
| *Porphyromonas* (OTU 7) | 39 | 34 | 2407 | 2 | 74 | 36 | 3 | 275 | 121 | 1 |
| *Streptococcus* (OTU 11) | 302 | 40 | 192 | 886 | 132 | 144 | 283 | 55 | 166 | 649 |
| *Fusobacterium* (OTU 9) | 1372 | 12 | 838 | 11 | 307 | 64 | 31 | 95 | 98 | 2 |
| *Neisseria* (OTU 15) | 62 | 125 | 631 | 17 | 463 | 84 | 32 | 66 | 615 | 207 |
| *Granulicatella* (OTU 8) | 84 | 288 | 255 | 42 | 124 | 938 | 79 | 73 | 41 | 17 |
| *Alloprevotella* (OTU 14) | 3 | 1 | 608 | 0 | 80 | 305 | 8 | 14 | 9 | 0 |
| *Corynebacterium* (OTU 46) | 878 | 0 | 0 | 0 | 1 | 1 | 0 | 0 | 2 | 0 |
| *Fusobacterium* (OTU 141) | 617 | 9 | 101 | 7 | 10 | 3 | 4 | 36 | 46 | 0 |
| *Lactobacillus* (OTU 13) | 1 | 0 | 0 | 751 | 0 | 0 | 2 | 0 | 0 | 0 |
| *Fusobacterium* (OTU 22) | 198 | 7 | 320 | 7 | 118 | 14 | 18 | 30 | 28 | 0 |
| *Actinomyces* (OTU 35) | 464 | 4 | 7 | 8 | 10 | 12 | 6 | 13 | 156 | 2 |
| unclassified (OTU 10) | 5 | 0 | 664 | 1 | 0 | 2 | 0 | 0 | 2 | 0 |
| *Haemophilus* (OTU 17) | 0 | 100 | 55 | 4 | 330 | 6 | 1 | 18 | 57 | 47 |
| *Actinomyces* (OTU 31) | 112 | 7 | 208 | 5 | 66 | 66 | 18 | 93 | 22 | 8 |
| *Leptotrichia* (OTU 37) | 477 | 0 | 4 | 2 | 0 | 0 | 11 | 6 | 1 | 2 |
| *Leptotrichia* (OTU 36) | 425 | 0 | 2 | 7 | 0 | 2 | 1 | 1 | 1 | 0 |
| *Lautropia* (OTU 16) | 120 | 23 | 140 | 2 | 88 | 25 | 0 | 2 | 24 | 9 |
| *Streptococcus* (OTU 27) | 142 | 0 | 0 | 211 | 0 | 0 | 0 | 4 | 1 | 0 |
| *Lachnoanaerobaculum* (OTU 30) | 196 | 2 | 49 | 0 | 16 | 42 | 12 | 26 | 4 | 1 |
| *Rothia* (OTU 25) | 114 | 100 | 11 | 13 | 16 | 34 | 4 | 12 | 29 | 9 |
| *Capnocytophaga* (OTU 99) | 304 | 0 | 2 | 1 | 1 | 0 | 0 | 0 | 1 | 0 |
| *Leptotrichia* (OTU 87) | 285 | 1 | 3 | 0 | 1 | 1 | 0 | 3 | 1 | 2 |
| *Campylobacter* (OTU 47) | 287 | 0 | 1 | 1 | 0 | 0 | 0 | 2 | 2 | 0 |
| *Capnocytophaga* (OTU 29) | 178 | 1 | 9 | 1 | 3 | 0 | 2 | 6 | 90 | 0 |
| *Capnocytophaga* (OTU 55) | 258 | 0 | 1 | 1 | 2 | 0 | 1 | 0 | 22 | 0 |
| *Veillonella* (OTU 19) | 0 | 13 | 56 | 5 | 0 | 0 | 1 | 188 | 0 | 5 |
| *Aggregatibacter* (OTU 64) | 40 | 1 | 7 | 4 | 0 | 0 | 0 | 0 | 200 | 0 |
| *Campylobacter* (OTU 84) | 240 | 0 | 2 | 1 | 0 | 2 | 0 | 3 | 4 | 0 |
| unclassified (OTU 48) | 239 | 0 | 0 | 0 | 0 | 0 | 0 | 0 | 0 | 0 |
| *Atopobium* (OTU 45) | 139 | 0 | 39 | 2 | 12 | 15 | 5 | 9 | 1 | 0 |
| *Capnocytophaga* (OTU 24) | 8 | 0 | 6 | 0 | 1 | 0 | 1 | 0 | 196 | 1 |
| unclassified (OTU 12) | 8 | 9 | 138 | 1 | 0 | 0 | 0 | 44 | 1 | 2 |
| *Streptococcus* (OTU 42) | 191 | 0 | 0 | 0 | 0 | 0 | 0 | 0 | 2 | 0 |
| *Dialister* (OTU 44) | 186 | 0 | 1 | 1 | 0 | 0 | 0 | 0 | 0 | 0 |
| *Aggregatibacter* (OTU 21) | 37 | 0 | 103 | 1 | 0 | 3 | 1 | 6 | 32 | 3 |
| *Olsenella* (OTU 63) | 185 | 0 | 0 | 0 | 0 | 0 | 0 | 1 | 0 | 0 |
| *Lactobacillus* (OTU 51) | 159 | 0 | 0 | 0 | 0 | 0 | 0 | 0 | 0 | 0 |
| unclassified (OTU 57) | 130 | 1 | 15 | 1 | 0 | 1 | 0 | 5 | 4 | 0 |
| *Lactobacillus* (OTU 39) | 25 | 0 | 0 | 124 | 0 | 0 | 0 | 0 | 1 | 0 |
| unclassified (OTU 43) | 150 | 0 | 0 | 0 | 0 | 0 | 0 | 0 | 0 | 0 |
| *Capnocytophaga* (OTU 67) | 121 | 0 | 7 | 1 | 0 | 0 | 3 | 1 | 8 | 0 |
| *Leptotrichia* (OTU 70) | 103 | 0 | 25 | 1 | 3 | 2 | 0 | 3 | 4 | 0 |
| *Lactobacillus* (OTU 41) | 27 | 0 | 0 | 103 | 0 | 0 | 0 | 0 | 0 | 0 |
| unclassified (OTU 54) | 126 | 0 | 0 | 0 | 0 | 1 | 0 | 1 | 0 | 0 |
| unclassified (OTU 188) | 120 | 0 | 0 | 0 | 0 | 0 | 0 | 0 | 0 | 0 |
| *Actinomyces* (OTU 59) | 115 | 0 | 0 | 0 | 0 | 0 | 0 | 0 | 0 | 0 |
