# Supplementary figures and images for: Visualizing the dental biofilm matrix by means of fluorescence lectin-binding analysis
Source: J Oral Microbiol. 2017 Jul 9;9(1):1345581. doi: 10.1080/20002297.2017.1345581 (PMC5508396; doi:10.1080/20002297.2017.1345581)

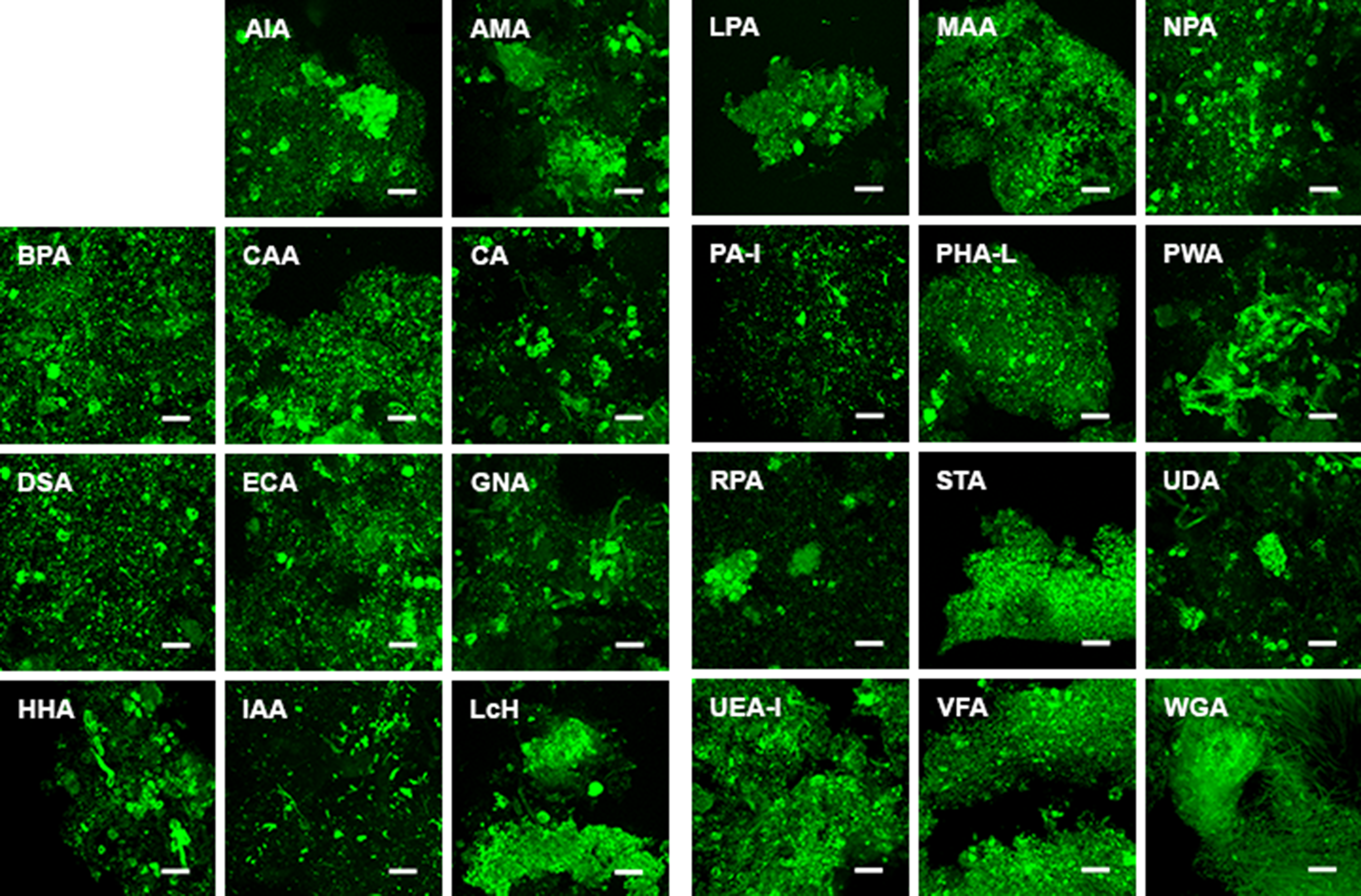

Supplement: Supplemental_data.zip [file zjom_a_1345581_sm2449.zip › Supplemental data/figure S2_corr.tif]

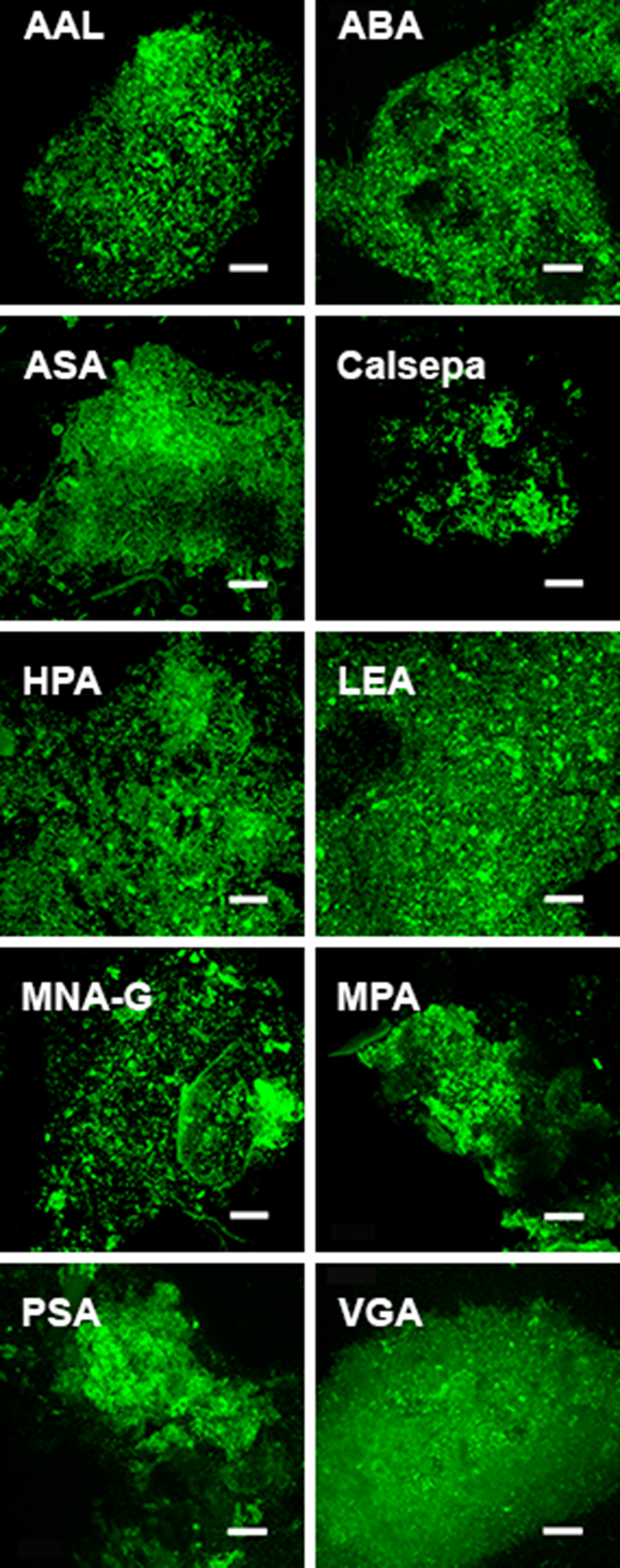

Supplement: Supplemental_data.zip [file zjom_a_1345581_sm2449.zip › Supplemental data/Suppl__3.tif]
